# Modeling Gross Primary Production of Agro-Forestry Ecosystems by Assimilation of Satellite-Derived Information in a Process-Based Model

**DOI:** 10.3390/s90200922

**Published:** 2009-02-13

**Authors:** Mirco Migliavacca, Michele Meroni, Lorenzo Busetto, Roberto Colombo, Terenzio Zenone, Giorgio Matteucci, Giovanni Manca, Guenther Seufert

**Affiliations:** 1 Remote Sensing of Environmental Dynamics Lab., DISAT, University of Milano-Bicocca, Milano, Italy; 2 Institute on Atmospheric Pollution (IIA), National Research Council (CNR), Roma, Italy; 3 Department of Forest Science and Environment, University of Tuscia, 01100 Viterbo, Italy; 4 European Commission, DG-JRC, Institute for Environment and Sustainability, Climate Change Unit, Ispra (VA), Italy; 5 Institute for Mediterranean Agricultural and Forest Systems, National Research Council, Rende (CS), Italy

**Keywords:** Gross Primary Production, Phenology, BIOME-BGC, PROSPECT, SAILH, Poplar plantations

## Abstract

In this paper we present results obtained in the framework of a regional-scale analysis of the carbon budget of poplar plantations in Northern Italy. We explored the ability of the process-based model BIOME-BGC to estimate the gross primary production (GPP) using an inverse modeling approach exploiting eddy covariance and satellite data. We firstly present a version of BIOME-BGC coupled with the radiative transfer models PROSPECT and SAILH (named PROSAILH-BGC) with the aims of i) improving the BIOME-BGC description of the radiative transfer regime within the canopy and ii) allowing the assimilation of remotely-sensed vegetation index time series, such as MODIS NDVI, into the model. Secondly, we present a two-step model inversion for optimization of model parameters. In the first step, some key ecophysiological parameters were optimized against data collected by an eddy covariance flux tower. In the second step, important information about phenological dates and about standing biomass were optimized against MODIS NDVI. Results obtained showed that the PROSAILH-BGC allowed simulation of MODIS NDVI with good accuracy and that we described better the canopy radiation regime. The inverse modeling approach was demonstrated to be useful for the optimization of ecophysiological model parameters, phenological dates and parameters related to the standing biomass, allowing good accuracy of daily and annual GPP predictions. In summary, this study showed that assimilation of eddy covariance and remote sensing data in a process model may provide important information for modeling gross primary production at regional scale.

## 1. Introduction

Terrestrial ecosystems play an important role in the global carbon cycle due to their capacity to sequester part of the fossil carbon emitted by anthropogenic activities. Such a capacity is of great interest in order to comply with the commitments of the Kyoto Protocol.

In the context of global climate change and sustainable development, agro-forestry management activities play a key role through mitigation. However, agro-forestry ecosystems are also affected by climate change and their contribution to carbon sequestration may be influenced by stresses (e.g. heat-waves, drought, diseases and natural disturbances) [1]. Fast growing forests [2], and in particular poplar plantations, are a typical land-use in the Lombardy region (Northern Italy) covering about 4.5% of the agricultural area and 6.5% of the forestry area [3]. It is therefore relevant to investigate the sink capacity of these ecosystems, both at site level with field measurements and at regional scale with modeling tools.

In recent years, a number of process-based models have been developed for estimating carbon and water fluxes at different spatial and temporal scales [4,5]. Among these, BIOME-BGC [5,6] is a widely employed ecosystem model designed to simulate plant physiological processes and soil biogeochemistry with a very detailed scheme and at a fine temporal scale (from daily to yearly). BIOME-BGC has been applied with success to different types of forest ecosystems, from Mediterranean [7] to coniferous species [6,8]. However, to our knowledge, it is rare to find applications on agricultural [9] and agro-forestry ecosystems such as poplar plantations. Spatial analysis was also proven feasible in recent studies in which BIOME-BGC was successfully applied to model net primary production [10] and gross primary production (GPP) at regional and continental scales [11,12].

Model parameterization and corroboration can benefit from the availability of continuous measurements of carbon, water and energy fluxes between ecosystems and the atmosphere with the eddy covariance (EC) technique [13]. Moreover, these measurements represent an important data source for the optimization of uncertain or unknown model parameters by using an inverse modeling approach [14]. In fact, when parameters cannot be measured directly, inverse modeling allows exploitation of one or more measurable model outputs (e.g. carbon fluxes) to optimize the values of one or more unknown model parameters (e.g. allocation ratios). This optimization is performed by retrieving the set of model parameters that minimizes the difference between simulated and observed data. Many inverse modeling approaches, such as Monte Carlo methods, gradient-based optimization algorithms, look-up tables and neural networks have been proposed in literature [15,16] and successfully applied in earth observation [17-19], biogeochemistry [20,21] and phenological studies [22,23].

Besides EC, remote sensing (RS) is another tool that can be exploited to gather spatially and temporally distributed information which is well suited for regional applications of process-based models. As a consequence, a number of techniques for the assimilation of RS time series at different spatial resolution into process-based models have been developed [24]. These techniques can be grouped into three main categories: i) determination of model initialization parameters (e.g. phenological parameters, leaf area index), ii) update of model state variables through direct ingestion of RS data (forcing) and, iii) estimation of state variables or model parameters through model inversion against RS data (recalibration or optimization).

The accuracy of modeled GPP is dependent on a correct parameterization of plant ecophysiology and stand characteristics (e.g. standing biomass and phenology) [25]. For regional scale mapping, such parameters are unknown and have to be determined for each spatial location. Stand characteristics strongly controlling the modeled carbon fluxes such as standing biomass or phenology [26], can be mapped with an appropriate analysis of RS time-series. This is not feasible for other ecophysiological parameters (e.g. C:N ratios, fraction of available nitrogen in leaves) which are instead assumed to be constant for a given species [10] or even plant-functional type (e.g. evergreen needleleaf forest, deciduous broadleaved forest, etc.) as described in several applications at continental level [12]. This assumption neglects the temporal and within-species variability of such parameters but allows their spatial determination through the use of a land use map.

In this paper we present a modeling study conducted at site level which represents the first step toward the analysis of the carbon budget of poplar plantations at regional scale.

According to the previous distinction of model parameters in two groups, species-dependent (ecophysiological parameters) and spatially variable (standing biomass and phenological parameters), we propose the following modeling scheme:

1. determine the ecophysiological parameters exploiting site level EC measurements;
2. determine the spatially variable parameters necessary for modeling poplar productivity over large areas through assimilation of RS data into the optimized BIOME-BGC.

More in detail in this paper we describe:

1. a modified version of BIOME-BGC (named PROSAILH-BGC) which was developed by coupling BIOME-BGC with the vegetation radiative transfer models PROSPECT and SAILH. The aims of this coupling were twofold: *i)* to improve the description of the radiative transfer regime within the canopy and *ii)* to allow assimilation of remotely-sensed vegetation indexes time series, such as MODIS NDVI, into the process-based model.
2. an inverse modeling approach developed for the optimization of the key [25] ecophysiological parameters of the PROSAILH-BGC. In this first-step optimization, model parameters were optimized for poplar plantations by inverting the model against EC data measured at the experimental field site.
3. a technique developed for assimilation of MODIS NDVI data into the process model. For this purpose we inverted the PROSAILH-BGC against the MODIS NDVI (second-step optimization) in order to retrieve key drivers [25] of modeled GPP (e.g. start and end of growing season, maximum leaf carbon during the year).
4. the evaluation of model accuracy: daily and yearly GPP modeled after two-step optimization were compared to site observations.

## 2. Data

### 2.1. Experimental Field Site Information

The study site is a managed poplar plantation located in a flat area periodically subjected to flooding near the village of Zerbolò in northern Italy (45° 12′ 03.14″ N, 9° 03′ 39.74 E, 60 m a.s.l.). The climate of the site is classified as Humid Subtropical - Mid Mild Latitude (Cfa) - Koppen Climate Classification [26], with yearly average rainfall of 912 mm and mean temperature of 12.5°C. The site is characterized by high total nitrogen depositions of about 20 kgN ha^-1^ per year [27]. The soil texture is sandy-loam (60.4% sand, 30% silt and 9.6% clay). The nitrogen and carbon content of the soil, measured to a depth of 100 cm, are 1.36 kgN m^-2^ and 5.23 kgC m^-2^, respectively.

A 27-m scaffold tower was erected in March 2002 in the homogeneous stand of about 46 ha and disassembled in 2005, immediately before logging. The EC flux tower, belonging to the Carboeurope-IP network (site ID: IT-PT1), measured continuously carbon, water and energy fluxes between ecosystems and atmosphere. The plantation was characterized by a spacing of 6 × 6 m and a tree density of 278 trees ha^-1^. Mean tree height, mean diameter at breast height (DBH) and the stem basal area, measured in 2005, were 26.3 (± 4.5) m (n = 8), 32.9 (± 5.7) cm (n = 266) and 20.45 m^2^ha^-1^, respectively. The leaf area index (LAI) was measured during the growing season every two weeks with LAI-2000 PCA plant canopy analyzer (LI-COR Inc., Lincoln NE, USA) as described in [28]. The LAI showed a seasonal variability with a maximum value of about 2.0 m^2^m^-2^ reached generally in July. The mean specific leaf area (SLA) of poplar leaves was 12.3 (± 1.8) m^2^kgC^-1^. SLA was estimated by extracting a known sub-area from the leaves collected during the growing season. Three leaves, at two different canopy levels (bottom and top), from three plants around the flux tower and from three plants located in the nearby stand were sampled. The leaves were collected every month from May to August for a total of five sampling dates.

The maximum taproot length reached about 1.5-1.7 m [29], while the soil depth was limited by the position of the water table at about 2.0 m.

### 2.2. Micrometeorological Data

A standard EC setup was used to collect micrometeorological data. Carbon and water fluxes were calculated with a time step of 30 minutes according to EUROFLUX methodology [30].

The CO_2_ fluxes were corrected and filtered following [30] in order to assess the quality of measured data and to discard the half-hourly data not fulfilling the hypothesis necessary for the application of the EC technique (i.e. steady state and integral turbulence characteristics of the vertical wind [31]). Data were corrected for storage of CO_2_ in the air layer below the measuring height [32]. Missing half-hourly data caused by malfunctioning of system, periodical calibration of instruments, u* filtering or data quality check not fulfilled, were filled with the marginal distribution sampling method [33].

EC measures the half hourly net ecosystem exchange of CO_2_ (NEE). Half-hourly GPP can be estimated from NEE via the general equation:

(1) $$\text{GPP}=-\text{NEE}+R_{\text{eco}}$$

Ecosystem respiration (R_eco_), was estimated using the partitioning method described in [33]. Both the MDS and the partitioning algorithm were implemented in an online tool [33], widely employed by the Carboeurope-IP project and FLUXNET network for both gap-filling and partitioning [34,35] of fluxes.

Along with the flux measurements, standard micro-meteorological data were collected continuously. Moreover, PAR transmitted below the canopy at ground level (PAR_t_) was measured by means of a transect of 3 quantum sensors (LI-190S, LI-COR Inc., Lincoln NE, USA) and used to investigate the radiative regime within the canopy. All meteorological data were stored as half-hourly means on a data logger (DL2 DELTA-T Devices, Burwell, Cambridge, UK).

### 2.3. Remotely Sensed Data

MODIS 250m 16-day composite NDVI data acquired by the TERRA platform (Product MOD13Q1) were downloaded from the Earth Observation System (EOS) data gateway. MODIS NDVI temporal profiles (NDVI_MODIS_) were affected by errors related to the presence of cloudy sky conditions over the compositing period. In order to reduce this influence and to reconstruct high-quality vegetation index time series, the original MODIS NDVI time series were processed following the method proposed by [36], which is based on the recursive application of a Savitzky-Golay filter [37]. The smoothed MODIS NDVI time series of the studied poplar plantation were then extracted as the average of the three pixels within the experimental field.

## 3. Methods

### 3.1. BIOME-BGC Description

BIOME-BGC is a mechanistic ecosystem model that simulates biogeochemical and hydrologic processes of terrestrial ecosystems based on the assumption that differences in the process rates are a function of climate and general life-form characteristics. BIOME-BGC simulates carbon, nitrogen and water cycles within a forest ecosystem across several compartments (including leaf, root, stem, soil and atmosphere) [5]: the main processes described by the model are evapotranspiration (Penman–Monteith equation), photosynthesis (Farquhar model, [38]) and respiration, modeled as the sum of autotrophic respiration [39] and heterotrophic respiration.

NEE (kgC m^-2^ day^-1^) was modeled as the net accumulation or loss of carbon by the entire soil-stand system and was determined by the differences between GPP (kgC m^-2^ day^-1^), resulting from the processes of photosynthesis, and R_eco_ (kgC m^-2^ day^-1^), resulting from the respiration processes. The LAI (m^2^ m^-2^) is a key variable of BIOME-BGC controlling canopy radiation absorption, water interception, photosynthesis and litter input to detrital pools [40].

The model requires three types of information: ecophysiological parameters, site parameters and meteorological data. The main ecophysiological properties include the carbon to nitrogen ratios of the different plant pools (e.g. carbon to nitrogen leaf ratio, C:N_leaf_), carbon allocation parameter (e.g. new fine root to new leaf carbon, FR:LC), maximum stomatal conductance (g_c,MAX_), canopy water interception, light extinction coefficients and SLA.

The main site parameters include soil depth, soil texture, initial standing biomass (e.g. maximum leaf carbon during the simulated year, LC_MAX_ and maximum stem carbon) and initial soil carbon in the different soil pools. The model also requires the bud-burst date (ONDAY), in which the growing season starts, and the day of the end of growing season (OFFDAY). These phenological parameters strongly influence the seasonal pattern and magnitude of simulated carbon fluxes [25].

With regards to meteorological data, the model requires the maximum, minimum and mean daily air temperature, mean daylight VPD, daily precipitation, mean daily incoming shortwave radiation and day-length.Model outputs include GPP, NEE, evapotranspitration and LAI simulated at a daily time-step. We used version 4.1.1 of the BIOME-BGC code available on the web site of the Numerical Terradynamics Simulation Group (NTSG) of the University of Montana. More information on the design of the model and its functioning can be found in [6, 25, 40].

### 3.2. PROSAILH-BGC Description

BIOME-BGC was coupled with the leaf and canopy radiative transfer (RT) models named PROSPECT and SAILH [19], respectively, resulting in the coupled model referred to hereafter as PROSAILH-BGC. The objectives of the coupling were twofold: to improve the BIOME-BGC description of the canopy radiation regime and to simulate the NDVI as a function of LAI and overpass characteristics (sensor and sun geometry) of MODIS observations.

BIOME-BGC computes net shortwave radiation using a constant surface albedo during the year. This assumption is violated in deciduous ecosystems where the albedo is closely related to foliar phenology [41]. Moreover, the radiation transmitted and absorbed by the canopy is modeled according to Lambert-Beer's law. The radiation absorbed by the canopy is therefore a function of the incoming PAR radiation, the LAI and of biome-specific canopy light extinction coefficients. The Lambert-Beer formulation neglects multiple scattering in the vegetated medium. To overcome such limitations we replaced the RT subroutine of the model with the PROSAILH model. PROSAILH is a combination of two models: PROSPECT [42], which describes leaf optical properties and SAILH [43], which computes top-of-the-canopy spectral bidirectional reflectance.

PROSPECT is a plate model that simulates reflectance and transmittance of a leaf as a function of four state variables: leaf structure parameter, chlorophyll a+b concentration, water content and leaf mass area.

SAILH is a one-dimensional bidirectional turbid medium RT model that simulates canopy bidirectional reflectance for a given sun-sensor geometry, canopy background reflectance, LAI, mean leaf inclination angle, hot-spot-size parameter and background brightness factor. The choice of SAILH is justified by its theoretical simplicity and advantages for application purposes. Nevertheless, this model assumes a dense canopy, which may not be the case with young poplar plantations. Sparse canopies in fact originate shadowing (between crowns and on the soil background) which are not simulated by the RT model. Shadowing effects were minimized by working with the vegetation optical index NDVI. Moreover, it must be noted that when using MODIS data at 250 m spatial resolution (so-called medium resolution) the 1D approach can accurately represent the reflected, transmitted and absorbed fraction of vertical fluxes, irrespective of local variability exhibited by the canopy at a finer spatial scale [18]. On the contrary, the retrieved variables (e.g. LAI) are the domain-average effective variables instead of the actual ones. We believe that this is not a major problem because these effective variables are used to feed BIOME-BGC, which is indeed a simplification of the real world, using effective variables as well.

The PROSAILH requires a total of eight input parameters (Table 1). LAI was provided with the daily step by BIOME-BGC, while the other input parameters were kept constant and parameterized according to a previous study on poplar plantations [17]. Fixing leaf parameters to constant values represented a simplification of the RT problem because of leaf age and darkening during the growing season. Another simplification that was applied regards the fact that our modelling approach does not include the understory vegetation that may be present in poplar plantations. Both natural processes (changes of leaf optical properties, understory growth) are to some extent picked up by NDVI, but neglected in our modelling approach.

**Figure 1.**
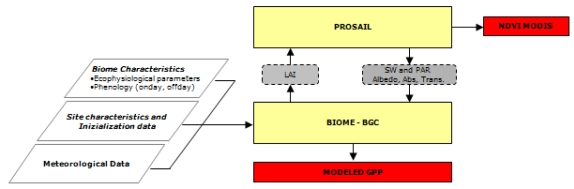
Flow chart of the PROSAILH-BGC model. Yellow blocks represent the models, parallelepipeds represent the input parameters, grey boxes represent the state variables passed between the coupled models, while the red boxes are the model outputs (NDVI and GPP).

The PROSAILH-BGC flowchart is reported in Figure 1. Daily LAI, simulated by the BIOME-BGC, is used as input for the PROSAILH which simulates NDVI, daily shortwave radiation, PAR_t_, PAR absorbed by the canopy and site albedo. These variables are then forced into the BIOME-BGC and used for daily simulations.

The additional key output of the PROSAILH-BGC with respect to BIOME-BGC is the daily NDVI (NDVI_PROSAILH-BGC_), which is computed using simulated canopy spectral reflectance taking into account MODIS spectral characteristics and sun-sensor geometry.

## 3.3. Basic Model Parameterization

The BIOME-BGC was parameterized using information about vegetation and site characteristics and used as a reference model for the comparison with the modified one. Daily meteorological data were measured at the experimental site, while soil and stand characteristics were obtained from [29,44]. Standing biomass data (LC_MAX_ and first-year maximum stem carbon) were obtained from a specific stand characterization conducted during the years 2002 and 2003. We avoided the “spin up and go” mode, which initializes site characteristics and finds an internal equilibrium (i.e. steady-state) of the model state variables [25], because poplar plantations are typical managed and disturbed ecosystems far from the steady-state. With the exception of SLA, which was measured on site, all the ecophysiological parameters required by BIOME-BGC were derived from literature [10,25,45,46] and are reported in Appendix I.

We defined two reference models to be compared with the optimized one. In both, the ecophysiological parameterization was derived from literature while phenology (ONDAY, OFFDAY) was parameterized using different methods: for Reference Model 1, the dates were defined with the BIOME-BGC internal phenological model while in the Reference Model 2 the dates were determined as the dates of the inversion (positive to negative and viceversa) of the 10-day running average of the measured NEE.

### 3.4. PROSAILH-BGC Optimization

An inverse modeling approach was used to recalibrate the input parameters of the PROSAILH-BGC model. The optimization technique [47] was based on a Truncated-Newton method [48] that minimizes iteratively the cost function (C) with respect to a given set of model parameters *θ*. The C defined for this study was the normalized least squared differences between observed and modeled data:

(2) $$C(\theta )=\frac{1}{n}\sum_{i=1}^{n}{\left(\frac{obs_{i}-{\text{mod}}_{i}(\theta )}{obs_{i}}\right)}^{2}$$

where *n* is the number of observations, *obs_i_* and *mod_i_* indicate the *i*^th^ observed and *i*^th^ modeled daily data, respectively. *mod* is a function of the parameter vector *θ*.

**Figure 2.**
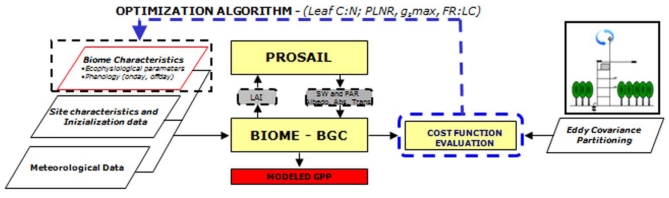
Flow chart of first-step optimization. Yellow blocks represent the models, parallelepipeds represent the model input parameters and the data for model optimization, grey boxes represent the state variables passed between coupled models while the red box is the model output.

**Figure 3.**
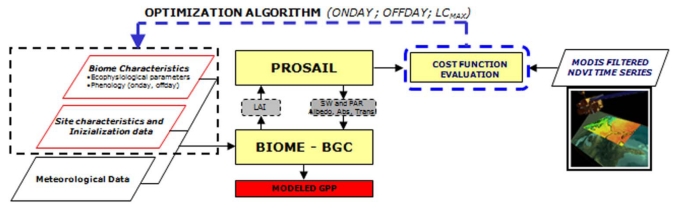
Flow chart of second-step optimization. Yellow blocks represent the models, parallelepipeds represent the model input parameters and the data for model optimization, grey boxes represent the state variables passed between coupled models while the red box is the model output.

The optimization strategy was based on a two-step approach:

1. In the first step the model was optimized against GPP observations to estimate the ecophysiological parameters (Figure 2) for poplars for a further large-scale application. The target variables selected for optimization were C:N_Leaf_, the percentage of leaf nitrogen in RUBISCO (PLNR), FRC:LC and g_s,MAX_. We selected these parameters because they exert a significant influence on the modeled carbon fluxes, as pointed out by the sensitivity analysis described in [25]. In this step phenological observations (ONDAY, OFFDAY) and LC_MAX_ were fixed to the observed values.
  Model ecophysiological parameters and their relative standard errors were estimated by using a bootstrapping algorithm with N = 500 resampling as described in [49]. The median of the distribution generated by bootstrapping for each parameter represents the estimated parameter value, while the standard deviation is a good measure of the error associated with the parameters.
2. In the second step we estimated phenological and standing biomass related parameters by inverting the model against remotely sensed NDVI time series. The algorithm determines ONDAY, OFFDAY and LC_MAX_ which minimize the cost function calculated using NDVI_MODIS_ as observation and the NDVI_PROSAILH-BGC_ as modeled data (Figure 3). These parameters were chosen because of their importance for the model application at spatial scale. In fact, process-based models, and in particular BIOME-BGC, are sensitive to parameters describing the development of the canopy such as phenological data and parameters related to maximum LAI [25]. Thus, in this step we evaluate the accuracy of the proposed method in retrieving these important data, usually lacking over large areas.

Even though ecophysiological parameters and stand characteristics can be correlated, we assumed that they could be estimated independently in two subsequent optimizations by exploiting two different data sources. In the first step, stand characteristics are set to their measured values. Thus, the optimized ecophysiological parameters may be considered as representative for the examined species and not a model inversion artifact due to an uncorrected parameterization of stand characteristics.

### 3.5. Evaluation of Model Accuracy

The accuracy of the modeled data can be evaluated by means of different statistics as described in [50]. In this study we used RMSE and modeling efficiency (EF) [51]. Slope (b[0]), intercept (b[1]) and r^2^ of the linear regression observed *vs* modeled were also used for the evaluation of model accuracy.

For correct model corroboration, simulations obtained with a specific set of parameters should be evaluated against a completely independent dataset, whose availability is usually rare in the case of environmental models. With regard to first-step optimization, we optimized the ecophysiological parameters against eddy covariance data observed during 2002 and corroborated the optimized model against data measured in 2003.

For second-step optimization, we firstly evaluated the accuracy of modeled NDVI time series. Secondly, we compared the retrieved ONDAY, OFFDAY and LC_MAX_ with the observed ones. Finally, we evaluated the error in the annual GPP budget introduced using the proposed method and the BIOME-BGC internal phenological routine. As an overall evaluation of the proposed method, the observed GPP and the GPP modeled with PROSAILH-BGC optimized in the first step and using the phenological dates and the LC_MAX_ derived from the second step were compared.

## 4. Results and Discussion

### 4.1. Radiative Regime Description of PROSAILH-BGC

**Figure 4.**
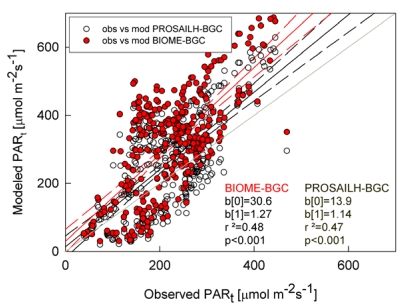
Relationship between modeled and observed PAR_t_. Red circles represent data modeled with BIOME-BGC while white circles represent data modeled with PROSAIL-BGC. Dashed lines represent the 95% confidence intervals of the linear regression between PAR_t_ modeled (with BIOME-BGC in red and PROSAIL-BGC in black) and observed data. Grey line is the 1:1 line. b[0] is the intercept, blsqb;1] is the slope and *p* is the significance of the linear regression analysis.

The performances of PROSAILH-BGC and BIOME-BGC in describing the radiative transfer regime within the canopy were tested by comparing the observed and modeled PAR_t_ with the two models. Results showed a reduction in the bias of PAR_t_ using the PROSAILH-BGC (Figure 4). In fact, although the r^2^ did not improve using PROSAILH-BGC, PAR_t_ simulated with the coupled model was closer to the 1:1 line than the PAR_t_ modeled by BIOME-BGC, thus leading to a reduction in RMSE between modeled and observed data (from 144.9 μmol m^-2^s^-1^ to 111.2 μmol m^-2^s^-1^).

### 4.2. First-step Optimization - PROSAILH-BGC Eecophysiological Parameter Estimates

The model parameter estimates obtained through model optimization against GPP measured during 2002, their relative standard errors and statistics for the evaluation of model accuracy are listed in Table 2. The optimized parameters (θ_opt_) showed considerable differences with respect to the original literature-based parameterization (used for Reference Models 1 and 2). In particular, FRC:LC showed a sensible increase (from 0.333 to 1.969). This optimized value is consistent with values published for broadleaved species ([25] (with values from 0.54 to 1.59 found) and for other poplar species ([10] (for which a value of 1.2 was reported). This may therefore indicate that the original ecophysiological parameterization based on the works of [10,25,45,46] was unsuited for the investigated poplar species.

Linear regression analysis between observed and modeled data showed an increase in both determination coefficient (from 0.88 to 0.93), slope (from 0.68 to 0.83), EF (from 0.78 to 0.88) and a decrease in RMSE (from 2.31 to 1.12 gCm^-2^day^-1^), between the optimized values and the Reference Model 2. Cumulated yearly GPP for 2002 simulated with the optimized model was 1546 gC m^-2^ year^-1^, with good agreement with the measured data of 1578 gC m^-2^ year^-1^. Conversely, GPP simulated by PROSAILH-BGC with the original parameterization was 1362 gC m^-2^year^-1^, while yearly GPP simulated with the Reference Model 2 (i.e. literature ecophysiological parameterization and phenology derived from site observations) was 1330 gC m^-2^year^-1^, with an underestimation of about 248 gC m^-2^ year^-1^.

As a validation exercise, the accuracy of the optimized model was evaluated using the GPP measurements collected during 2003, achieving a good improvement in GPP estimation with respect to the results obtained by PROSAILH-BGC with the original literature parameterization. Although modeled GPP with the optimized and original parameters explained the same amount of variance of the observed GPP (r^2^ = 0.78 for both the models), the RMSE decreased from 1.81 gC m^-2^day^-1^ to 1.41 gC m^-2^day^-1^ and EF increased from 0.67 to 0.76 as a consequence of the optimization. This improvement in model accuracy underscores that the main effect introduced by the optimized parameters was the reduction of the bias with a reduction of the systematic underestimation of the model. Conversely, the correlation between observations and modeled data did not improve when using the optimized parameterization because the daily variability of simulated fluxes is mainly driven by meteorological data.

### 4.3. Second-Step Optimization - Phenological and Standing Biomass Parameter Estimates

**Figure 5.**
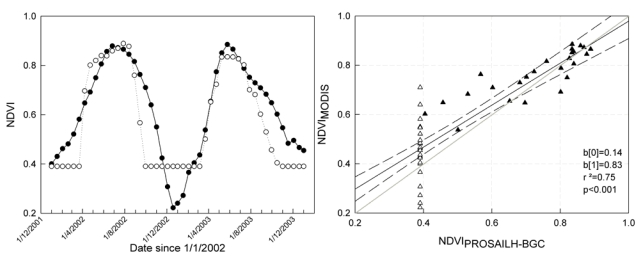
a) Time series of NDVI_MODIS_ (full circles) and NDVI_PROSAILH-BGC_ (open circles) for the time period 2002-2003. b) Scatterplot of NDVI_MODIS_ and NDVI_PROSAILH-BGC_. Black triangles are the NDVI data for the growing season (for the days between ONDAY and OFFDAY) while open triangles are data for the dormant period. The black straight line is the regression line calculated on the whole dataset, the dashed lines represent the 95 confidence intervals, the grey line is the 1:1 line. b[0rsqb; is the intercept, blsqb;1rsqb; is the slope and *p* is the significance of the linear regression analysis observed vs modeled.

The relationship between modeled and observed NDVI for the two growing seasons is reported in Figures 5a and 5b. NDVI_PROSAILH_ explained about 75% of NDVI_MODIS_ variance and showed good agreement between observed and modeled data (Figure 5b). For low NDVI values an underestimation of modeled NDVI was observed.

ONDAY, OFFDAY and LC_MAX_ estimated with second-step optimization for 2002 and 2003 showed good agreement with the values observed at the experimental site (Table 3).

With regard to ONDAY, we found 3 and 8 days of displacement between modeled and observed dates for 2002 and 2003, respectively, whereas for OFFDAY, the displacement was 7 and 6 days for 2002 and 2003, respectively. Conversely, the phenological dates estimated with the internal phenological model of BIOME-BGC led to higher offset between modeled and observed dates with an average displacement of 17 days for the ONDAY and 20 days for the OFFDAY. Moreover, LC_MAX_ values were estimated with good accuracy (mean error = 4.1%) as shown in Table 3.

The discrepancy between modeled and observed NDVI, as well as the offset between the estimated and observed phenological dates, may be due to the influence of the understory on the NDVI_MODIS_ signal, particularly noteworthy in the period immediately before the beginning and after the end of the growing season as shown in Figure 5a. However, these discrepancies between observed and modeled phenological dates are similar to those reported in others studies [52].

The effect of correct phenological parameterization on the determination of the annual GPP budget is reported in Table 4 where the yearly GPP estimated with the reference models and the two-step optimized model are listed. It is to be noted that by using the Reference Model 1 we obtained sensible underestimation of yearly GPP (-21% for the 2002, -26% for 2003). In Reference Model 2, in which the parameters related to phenology were set to the observed values, yearly GPP showed an underestimation of 15.7% for 2002 and 14.1% for 2003, with an improvement compared to Reference Model 1.

The error introduced using the internal phenological model is high even using the PROSAILH-BGC with the optimized ecophysiological parameters after first-step optimization (GPP_PROSAILH-BGC 1-step_); in fact, underestimation of the cumulated annual GPP was -10.4% for 2002 and -11.8% for 2003. The yearly GPP estimated after two-step optimization (GPP_PROSAILH-BGC 2-step_) showed good accuracy with an underestimation of 1.8% and 5.6% for 2002 and 2003, respectively. These results underline the importance of the phenological parameters in determining the annual GPP budget. Obviously, for application at regional scale, the parameterization of the model with the observed phenology is not operatively feasible. Hence, the proposed method may be considered an important option for determining these parameters.

**Figure 6.**
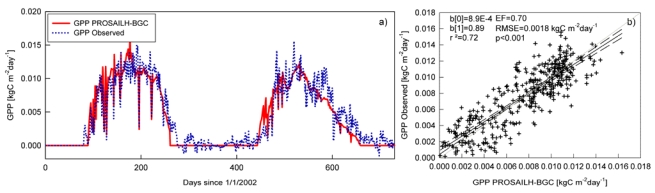
a) Time courses of modeled (red straight line) and observed (blue dotted line) GPP for 2002 and 2003. b) Scatterplot of observed and modeled GPP, data from both the growing seasons were plotted with exclusion of data of the dormant period. The black straight line is the regression line, the dashed lines represent the 95 confidence intervals, the grey line is the 1:1 line. blsqb;0rsqb; is the intercept, blsqb;1rsqb; is the slope and *p* is the significance of the linear regression analysis observed vs modeled.

Finally, we evaluated the accuracy of daily GPP modeled by PROSAILH-BGC after two-step optimization. The time courses of daily GPP for 2002 and 2003 are depicted in Figure 6a. Results show good agreement between modeled and observed GPP both at daily (Figure 6b) and yearly (Table 4) time-steps thus underscoring that the proposed approach may be useful in modeling the GPP of poplar plantations.

## 5. Summary and Conclusions

In this paper we present a modeling study conducted at site level which represents a first step toward the analysis of the carbon budget of poplar plantations at regional scale. We developed a PROSAILH-BGC coupling the RT Model PROSAILH with the BIOME-BGC model. This coupling allowed us to arrive at a more realistic description of the light regime within the canopy and to simulate the vegetation index NDVI, with the MODIS satellite sensor spectral and overpass-dependent characteristics, as a function of LAI provided by the ecosystem part of the model. This new feature of the model enabled us to assimilate high temporal frequency MODIS NDVI observations into the process-based model.

With this study we also provide a set of relevant ecophysiological parameters well suited for the application of PROSAILH-BGC for poplar plantations. The accuracy of the optimized model simulations, evaluated using an EC dataset not exploited in any calibration increased with respect to the PROSAILH-BGC applied using literature-based parameterization.

Modeled NDVI time series simulated MODIS data quite realistically (r^2^ = 0.75) and key phenological dates were retrieved with far better accuracy than the ones modeled by the internal phenological model: ONDAY and OFFDAY were determined with a mean error of 6 and 7 days, respectively, while with the internal phenological model the mean error was 17 days for ONDAY and 20 days for OFFDAY. The error in the dates estimated with second-step optimization may be due to the development of a green understory which affected the NDVI signal immediately before tree bud-burst and persisted after overstory leaf senescence. In the same computational step, maximum leaf carbon was also retrieved with an average error of 4.1%.

Globally, the two-step optimization process allowed fairly accurate estimates of GPP both at daily (r^2^ = 0.72, EF = 0.70, RMSE = 1. 80 gCm^-2^day^-1^) and yearly time steps. In particular, for the annual cumulated GPP we found a sensible reduction in the underestimation of modeled GPP after the two-step optimization compared to the results obtained using Reference Model 1, Reference Model 2 and also using the first-step optimized PROSAILH-BGC with phenology determined by the internal routine.

For the application of the model over large areas (e.g. regional scale), the unknown phenological parameters as well as the site structural parameters have to be specified in some way. These parameters set to a nominal value (e.g. based on few field observations) or modeled with the internal phenological routine may lead to errors in GPP estimations. This observation underlines the usefulness of the proposed procedure which provides a reliable estimate of such spatially variable parameters based on RS observations.

In summary, the proposed approach appears useful for modeling gross primary production both at site-level and at regional scale. In fact, we were firstly able to determine the correct species-dependent parameterization of the process-based model and, afterwards, we were able to assimilate remotely-sensed NDVI time series for the determination of spatially varying variables such as those related to phenology, canopy development and site standing biomass.

Further developments should be focused on the application of the method to different forest species and the development of a stable method for the optimization of other site characteristics required by the model (e.g. transfert growth and litterfall period; maximum stem carbon, soil effective depth), which are not easily available for application at the regional scale.

## Figures and Tables

**Table 1.** Parameterization of PROSAILH: leaf structure parameter (N), chlorophyll a+b concentration (C_AB_), leaf water content (C_w_), dry matter content (C_M_), Leaf Area Index (LAI) mean leaf inclination angle (θ_L_), hot spot size parameters (S_L_), the background brightness factor (α_S_). LAI is variable because it is estimated daily by BIOME-BGC.

***PROSAIL Parameters***		***Values***
N	-	1.37
C_AB_	μg cm^-2^	45
C_w_	g cm^-2^	0.0092
C_M_	g cm^-2^	0.0065
LAI	m^2^ m^-2^	variable
θ_L_	deg	56.5
S_L_	-	0.005
α_s_	-	1

**Table 2.** Original (*θ_or_*) and optimized (*θ_opt_*) parameters of the PROSAILH-BGC model. Standard errors of parameter estimates, calculated with the bootstrap algorithm, are shown in parentheses.

***Parameter***	***Unit***	***θ****_or_*	***θ****_opt_*
FRC:LC	-	0.333	1.969 (±0.420)
Leaf C:N	*kgC kgN^-1^*	15.59	20.93 (±2.50)
PLNR	-	0.088	0.1050 (±0.011)
g_s,MAX_	*m s^-1^*	0.006	0.0041 (±0.001)

**Table 3.** Start (ONDAY) and end (OFFDAY) of growing season, maximum leaf carbon (LC_MAX_) observed and estimated with second-step model optimization. The ONDAY and OFFDAY estimated with the internal phenological model (Internal phenology) were also reported. DOY is Day Of Year.

*Year*		*ONDAY*.	*OFFDAY*	*LC_MAX_*

		*DOY*	*DOY*	*kgCm^-2^*
2002	Obs.	91	267	0.164
	Second-step	88	260	0.159
	Internal phenology	100	289	-
2003	Obs.	78	315	0.155
	Second-step	70	309	0.147
	Internal phenology	107	297	-

**Table 4.** Annual GPP measured and simulated by BIOME-BGC with parameterization from literature and internal phenology (Reference Model 1), BIOME-BGC with parameterization from literature and observed phenology (Reference Model 2), by PROSAILH-BGC after first-step optimization with the internal phenology (*GPP_PROSAILH-BGC 1-step_*) and the final results obtained with PROSAILH-BGC after two step optimization.

*Year*	*GPP_measured_* *gC m^-2^yr^-1^*	*GPP_Reference Model 1_* *gC m^-2^yr^-1^*	*GPP_Reference Model 2_* *gC m^-2^yr^-1^*	*GPP_PROSAILH-BGC 1-step_* *gC m^-2^yr^-1^*	*GPP_PROSAILH-BGC 2-step_* *gC m^-2^yr^-1^*
2002	1,578	1,253	1,330	1,414	1,550
2003	1,473	1,084	1,265	1,299	1,391

## Appendix I

Ecophysiological parameterization of BIOME-BGC and PROSAILH-BGC as derived from literature for the Clone I-214. In grey the parameters involved in first-step optimization are outlined. For those parameters the value here reported is the one found in literature.

**Table:**

*ECOPHYSIOLOGICAL PARAMETERS - Clone I-214 (Populus x canadensis Moench)*		
78	(yday)	yearday to start new growth (when phenology flag = 0)
315	(yday)	yearday to end litterfall (when phenology flag = 0)
0.12	(prop.)	transfer growth period as fraction of growing season
0.38	(prop.)	litterfall as fraction of growing season
1.0	(1/yr)	annual leaf and fine root turnover fraction
0.70	(1/yr)	annual live wood turnover fraction
0.008	(1/yr)	annual whole-plant mortality fraction
0.0	(1/yr)	annual fire mortality fraction
1.2	(ratio)	(ALLOCATION) new fine root C: new leaf C
2.2	(ratio)	(ALLOCATION) new stem C: new leaf C
0.16	(ratio)	(ALLOCATION) new live wood C: new total wood C
0.22	(ratio)	(ALLOCATION) new croot C: new stem C
0.5	(prop.)	(ALLOCATION) current growth proportio
25.06	(kgC/kgN)	C:N of leaves
55.0	(kgC/kgN)	C:N of leaf litter, after retranslocation
42.0	(kgC/kgN)	C:N of fine roots
50.0	(kgC/kgN)	C:N of live wood
550.0	(kgC/kgN)	C:N of dead wood
0.38	(DIM)	leaf litter labile proportion
0.44	(DIM)	leaf litter cellulose proportion
0.18	(DIM)	leaf litter lignin proportion
0.34	(DIM)	fine root labile proportion
0.44	(DIM)	fine root cellulose proportion
0.22	(DIM)	fine root lignin proportion
0.77	(DIM)	dead wood cellulose proportion
0.23	(DIM)	dead wood lignin proportion
0.041	(1/LAI/d)	canopy water interception coefficient
0.54	(DIM)	canopy light extinction coefficient
2.0	(DIM)	all-sided to projected leaf area ratio
12.30	(m2/kgC)	canopy average specific leaf area (projected area basis)
2.0	(DIM)	ratio of shaded SLA:sunlit SLA
0.038	(DIM)	fraction of leaf N in Rubisco
0.006	(m/s)	maximum stomatal conductance (projected area basis)
6E-5	(m/s)	cuticular conductance (projected area basis)
0.01	(m/s)	boundary layer conductance (projected area basis)
-0.34	(MPa)	leaf water potential: start of conductance reduction
-2.2	(MPa)	leaf water potential: complete conductance reduction
1100.0	(Pa)	vapor pressure deficit: start of conductance reduction
3600.0	(Pa)	vapor pressure deficit: complete conductance reduction
